# Evaluation of a multidisciplinary neurological rehabilitation program for the post-COVID-19 condition

**DOI:** 10.1007/s00415-026-13693-5

**Published:** 2026-02-22

**Authors:** Marion Egger, Ralf Strobl, Lena Vogelgesang, Judith Reitelbach, Eva Grill, Klaus Jahn

**Affiliations:** 1 German Center for Vertigo and Balance Disorders, LMU University Hospital, LMU Munich, Munich, Germany; 2 Department of Neurology, Schoen Clinic Bad Aibling, Research Group, Kolbermoorer Str. 72, 8303 Bad Aibling, Germany; 3 Institute for Medical Information Processing, Biometry and Epidemiology, LMU Munich, Munich, Germany

**Keywords:** COVID-19, Post-acute COVID-19 Syndrome, Rehabilitation, Therapeutics, Neurological rehabilitation

## Abstract

**Objective:**

To evaluate a multidisciplinary therapy program with a neurological focus for individuals with post-COVID-19 condition, aiming to reduce symptom burden and improve functioning.

**Design:**

Non-experimental prospective before–after study.

**Subjects/patients:**

Individuals diagnosed with post-COVID-19 condition, defined as experiencing persistent signs and symptoms for more than 12 weeks after initial SARS-CoV-2 infection.

**Methods:**

We conducted a 2 week multidisciplinary rehabilitation program at the Schoen Clinic Bad Aibling, Germany. The intervention included multi-professional therapies. Assessments were conducted at six time points: baseline at the start of the 2-week control period, pre- and post-intervention, and at 2, 8, and 24 weeks post-intervention. Mixed-effects regression models were used to analyze changes over time. Outcome measures included health-related quality of life (HRQoL; EQ-5D-5L), fatigue, anxiety, depression, symptom severity, breathing difficulties, cognitive function, functional disability, and performance measures.

**Results:**

A total of 47 participants (60% female; mean age 49 years; range 21–80) were enrolled, with a median of 220 days (Q1: 156–Q3: 376) since initial infection. No significant improvement in HRQoL, grip strength, cognitive function, walking capacity, or balance was observed during the intervention compared to the control period. Fatigue, anxiety, depression, symptom severity, functional disability, and dyspnea improved significantly.

**Conclusion:**

This study indicates beneficial effects of a 2 week multidisciplinary therapy program on symptom burden and functional outcomes of the post-COVID-19 condition. Further research including randomized controlled trials is warranted.

**Trial registration:**

German Clinical Trials Register, DRKS00029415. Registered 04 July, 2022. Retrospectively registered. https://drks.de/search/en/trial/DRKS00029415

**Supplementary Information:**

The online version contains supplementary material available at 10.1007/s00415-026-13693-5.

## Introduction

COVID-19 is a multi-organ disease that can cause persistent symptoms, referred to as long-COVID after 4 weeks from infection and post-COVID-19 condition after 12 weeks from infection [1–3]. According to a large meta-analysis, 45% of COVID-19 survivors, both hospitalized and non-hospitalized patients, experienced at least one unresolved symptom approximately 4 months after infection [4]. These symptoms include fatigue, dyspnea, cough, muscle weakness, performance and activity limitations, and cardiovascular abnormalities [5–8]. Post-COVID-19 condition, as defined by the World Health Organization, represents a multisystem disorder rather than a specific neurological diagnosis, although neurological and neuropsychiatric functional limitations have been frequently reported. These include brain fog, memory issues, attention disorder, sleep disturbances, anxiety, depression, posttraumatic stress disorder, sensorimotor deficits, headache, and others [9–12]. In line with these symptoms and functional limitations, reduced health-related quality of life (HRQoL) and limitations in the ability to work and fulfill usual daily activities were reported [13–15]. Post-COVID-19 condition often persists, with symptoms reported up to 2 years after infection [16–18], with female sex, age, and education being important risk factors for delayed recovery [19]. Individuals who required hospitalization due to COVID-19 were reported to be at higher risk for post-COVID-19 condition compared to non-hospitalized individuals [20]. However, the post-COVID-19 condition can also occur after mild acute courses, and its manifestation can be even more severe than after critical acute cases [21]. The exact pathophysiology behind post-COVID-19 condition is still unknown, but biological mechanisms, including viral persistence, neuroinflammation, excessive blood clotting, and autoimmunity are discussed [22].

The devastating impact on daily life and the large number of affected individuals urgently necessitate the development of effective therapies. Rehabilitation programs, often with a pulmonary focus, have shown beneficial effects in reducing symptoms and improving functional exercise capacity, dyspnea, and HRQoL in individuals with post-COVID-19 condition [23–26]. Given the large number of neurological and neuropsychiatric symptoms, rehabilitation interventions with a neurological focus would be required. However, the effect of non-pharmacologic multifaceted interventions is still poorly understood, specifically for individuals with neurologic symptoms [27, 28].

Therefore, the objective of this study was to evaluate a multidisciplinary neurological therapy program for individuals with post-COVID-19 condition. We hypothesized that participation in the program would improve HRQoL and reduce post-COVID-19 symptom burden.

## Methods

### Study design, population and setting

We conducted an exploratory non-experimental prospective before–after study. Patients were recruited between June 2022 and October 2023 at the Schoen Clinic Bad Aibling, a center for neurorehabilitation in Germany. Study participants were invited through various channels: announcements in local newspaper articles, social media posts, information on the Schoen Clinic's homepage, and messages forwarded to general practitioners and neurologists in the vicinity.

The definition of post-COVID-19 condition of the World Health Organization was applied [3]. Adult patients (≥ 18 years) with a laboratory-confirmed SARS-CoV-2 infection verified by real-time reverse transcriptase PCR were eligible for inclusion if at least 3 months had passed since the acute infection and if they presented with persistent post-COVID-19 symptoms. Eligible neurological post-COVID-19 symptoms comprised cognitive impairment (“brain fog”), attention deficits, concentration deficits, memory difficulties, dizziness, balance disorders, headaches, and paresthesia. Non-neurological post-COVID-19 symptoms, which encompassed fatigue, exertional intolerance, dyspnea, palpitations, musculoskeletal pain, taste disturbance, gastrointestinal symptoms, mental health symptoms, and sleep disturbances, were permitted only in combination with at least one neurological symptom. All symptoms had to be of sufficient severity to reduce general health status or to limit activities of daily living. [29] Exclusion criteria were 1) terminal or other potentially life-threatening diseases preventing participation in an outpatient rehabilitation program, 2) requirement of inpatient care with need of monitoring and supervision, and 3) insufficient (German) communication skills to complete the questionnaires and take part in the therapies. No explicit exclusion criteria regarding pre-existing neurological conditions were applied.

**Fig. 1:**
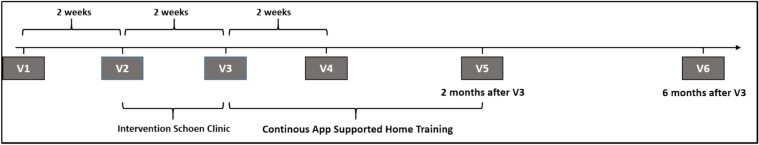
Timeline for study visits and interventions

Assessments were conducted in a cycle consisting of six study visits in complete (see Fig. 1 for an overview). The first study visit (V1) took part 2 weeks before the intervention period started at the Schoen Clinic. The next two study visits were completed at the beginning (V2) and the end (V3) of the 2-week intervention period (V4) was done 2 weeks after the end of the intervention. Two follow-up visits were done 8 weeks (V5) and 6 months (V6) after the end of the intervention period. The 2 weeks prior to the intervention period (V1–V2) served as the control period to observe changes in post-COVID symptoms. The study visits V1–V4 were conducted in person at the clinic; the follow-up visits (V5, V6) were conducted via telephone interviews and questionnaires sent by mail. Assessments were conducted by trained and experienced study staff between June 27, 2022, and March 31, 2023. The last follow-up interview was conducted on October 13, 2023.

The study was approved by the medical ethics committee of the Ludwig Maximilian University Munich according to the 1964 Declaration of Helsinki and its later amendments (project no. 22–0310). Written informed consent was obtained from all participants. The study was registered at the German Clinical Trials Register (DRKS00029415).

### Therapy interventions

The 2-week on-site intervention took place at various locations in the city of Bad Aibling, Bavaria, Germany, and at the Schoen Clinic Bad Aibling, a center for inpatient neurorehabilitation. The intervention was developed specifically for this study by an interdisciplinary team of local experts (physiotherapists, occupational therapists, neuropsychologists, physicians, and rehabilitation specialists). Its design was informed by clinical experience with post-COVID-19 patients and principles of neurorehabilitation, aiming to address physical, cognitive, and mental symptom domains while minimizing the risk of overexertion. The program was delivered 5 days per week, constituting a full-time, day-based intervention that included a mix of different rehabilitative interventions (e.g., Nordic walking, balance training, and cognitive training). It was a group-based rehabilitation program with a maximum of six participants who completed the 2-week intervention period together as a closed group. The rehabilitation program was primarily delivered in a group-based format, except for the peat bath and massage, cognitive computer training, Pablo training, and the individual neurologist and neuropsychologist consultation. A detailed description of the therapy interventions can be found in the Appendix (Supplementary Table 1). All participants were advised to pace themselves and take breaks to avoid overexertion [30]. A sample therapy schedule is provided in the Supplementary Information 1.

A subsequent 8-week digital app support for continuous home training was conducted from visit 3 on and included a digital therapy plan with exercise videos (e.g., balance and resistance training, stretching, breathing, and relaxation exercises) and educational videos (Caspar Health App (GOREHA GmbH, Germany; Supplementary Information 2).

### Outcome measures

Outcome measures included the following patient-reported outcomes and performance measures (a detailed description can be found in the Supplementary Information 3): EuroQol-5 dimensions-5 level (EQ-5D-5L; to measure health-related quality of life (HRQoL)) [31, 32], Fatigue Severity Scale-7 (FSS; to measure fatigue) [33], Hospital Anxiety and Depression Scale (HADS; to measure anxiety and depression) [34], World Health Organization Disability Assessment Schedule 2.0 (WHODAS-12; to measure overall disability) [35], Mini-Balance Evaluation Systems Test (Mini-BESTest; to measure balance problems) [36], hand grip strength, German version of the Montreal Cognitive Assessment (MoCA; to measure cognitive impairment) [37], modified Medical Research Council dyspnea scale (to measure dyspnea) [38], 2 min walk test (to measure functional capacity) [39], short form of DePaul Symptom Questionnaire (to measure myalgic encephalomyelitis and chronic fatigue syndrome symptomology) [40], modified COVID-19 Yorkshire Rehabilitation Screening test (C19-YRS; to measure specifically post-COVID-19 syndrome) [41], and the number of steps per day and resting heart rate both measured via smartwatch-based monitoring.

Satisfaction with the therapy intervention was evaluated using a self-designed questionnaire, based on the “Satisfaction with Therapy and Therapist Scale” [42].

### Confounding factors

Post-exertional malaise (PEM) is characterized by a worsening of symptoms following even minor physical or mental exertion that was tolerated previously and must be accounted for in rehabilitation programs, as it may negatively impact the rehabilitation outcome over time [43–45]. PEM was defined as the presence of next-day soreness following non-strenuous activity or physical fatigue after minimal exertion, as assessed by the items 2 and 3 of the DePaul Symptom Questionnaire at Visit 1 [40, 46].

Cognitive impairment was measured at V1 with a computer-based attention test [47]. We used the subtests Go/Nogo (inhibition ability and impulse control) and divided attention (ability to focus attention on two tasks simultaneously). Patients were classified as cognitively impaired if they scored below the 16th percentile in at least one of the subtests compared to normative values.

The subjective health before the COVID-19 infection was captured at V1 using the health score (0–10) of the C19-YRS.

### Statistical analysis

Summary statistics were calculated for the whole sample and separately for each study visit. Categorical variables were presented as absolute and relative frequencies, continuous variables as mean and standard deviation, and non-normally distributed continuous variables as median and interquartile range.

To examine the association between the intervention and the outcome measures, we applied longitudinal linear mixed-effects models with random intercepts and fixed slopes. The study design allowed us to use the 2-week period prior to the intervention (V1 to V2) as an internal control phase, during which no therapeutic exposure occurred. This phase provided an estimate of the natural fluctuation of post-COVID symptoms in the absence of treatment.

Time was modeled as a categorical variable representing each study visit, and intervention effects were evaluated by estimating the change in each outcome between consecutive visits. Thus, each time-point coefficient represents the adjusted mean difference in the outcome relative to the preceding visit, thus the coefficients quantify the change during the control period (V1 to V2), the first intervention week (V2 to V3), the post-intervention short-term period (V3 to V4), and so forth. A positive coefficient indicates an increase in the outcome compared with the prior visit, whereas a negative coefficient reflects a decrease. This modeling strategy allowed us to separate spontaneous symptom variation (control phase) from changes temporally associated with the intervention.

To account for potential confounding factors, all models were adjusted for age, sex, cognition, PEM, and pre-COVID-19 health status. In addition, we tested interaction terms between time and cognition as well as time and PEM to explore whether changes across study visits differed across these clinically relevant subgroups.

To assess multicollinearity, variance inflation factors (VIFs) were computed with a predefined threshold of points [48]. For each model, we report the intraclass correlation coefficient (ICC) as a measure of between-person clustering and the standard deviation of the random intercept as an estimate of between-individual variance [49].

To visualize the longitudinal effects of the intervention on the various endpoints, we used the results of the linear mixed-effects model to predict temporal progression. Specifically, we modeled the forecasted outcomes for a 49-year-old woman with PEM and cognitive impairment and visualized the respective trajectories together with the point-wise bootstrap confidence intervals. Predicted outcomes were plotted across the different study phases to illustrate the intervention’s impact over time.

Statistical analyses were performed using R version 4.3.2. A p-value ≤ 0.05 was considered significant. As this was an exploratory study aimed at understanding which aspects of the post-COVID-19 condition are influenced by the intervention, rather than evaluating the intervention as a whole, no correction for multiple testing was applied. Consequently, the p-values should be interpreted in conjunction with the respective effect estimates. Missing data were not imputed.

## Results

**Fig. 2:**
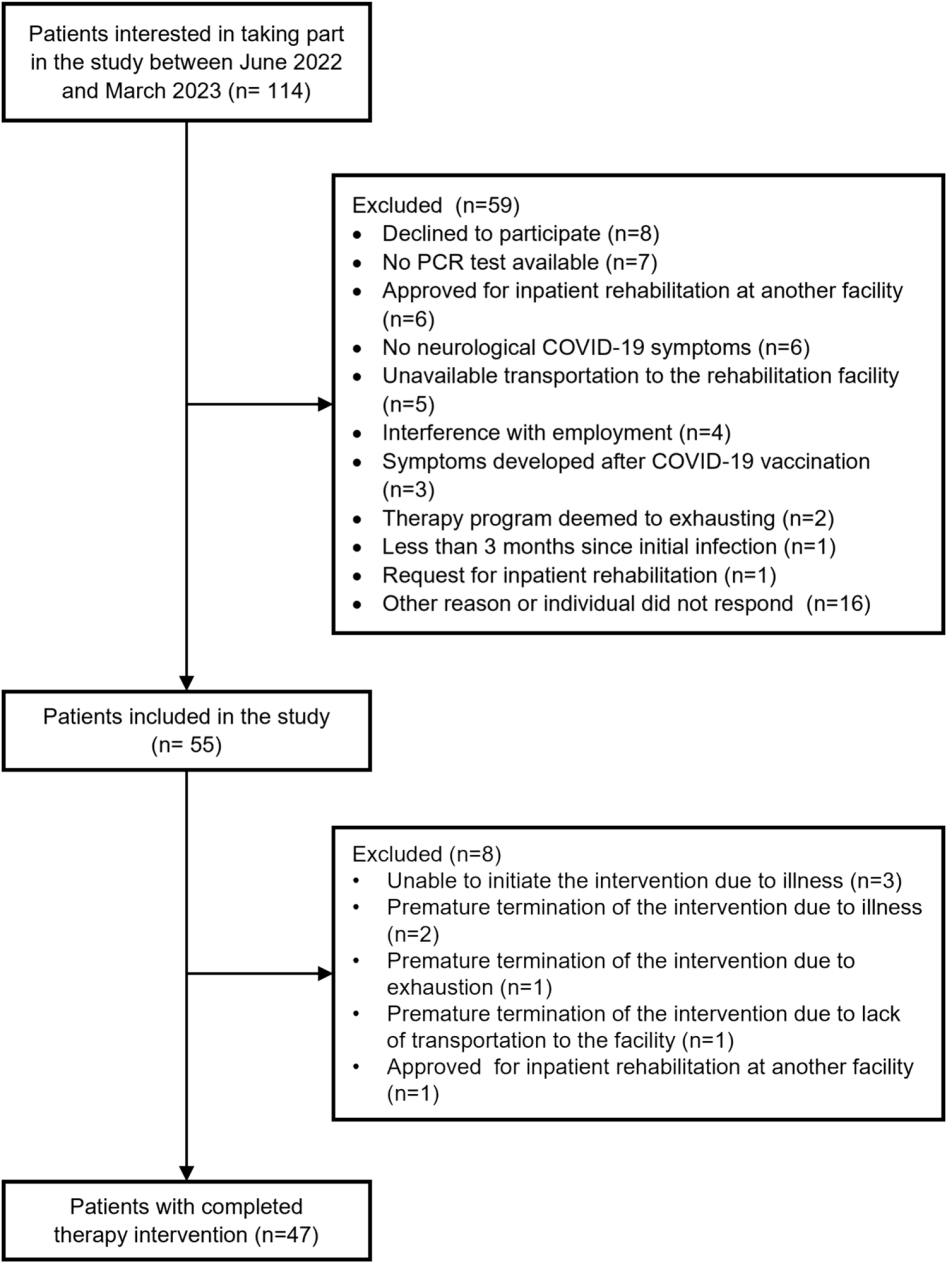
Flow chart showing participant enrollment

In total, 114 individuals were interested in taking part in the study and therefore contacted our study team. Of these, 55 persons were included in the study and 47 individuals (60% female, mean age 49 years) completed the entire therapy intervention within ten study circles (Flow chart in Fig. 2). Participant’s characteristics are displayed in Table 1.

**Table 1:** Characteristics of included complete cases at first study visit (*n* = 47)

Variable	Statistic*
Age at first infection, years	48.7 ± 15.8
Sex, women	28 (59.6)
Duration since first infection, days	220.0 (156.0—376.0)
Number of COVID-19 infections	
1	31 (66.1)
2	14 (29.8)
≥ 3	2 (4.3)
Vaccination at the time of first infection	
Complete vaccination	25 (53.2)
Partial vaccination	3 (6.4)
No vaccination	16 (34.0)
Missing	3 (6.4)
Hospitalized during COVID-19 infection (Yes)	2 (4.3)
Elixhauser Comorbidity Index	
< 0	3 (6.3)
= 0	38 (80.8)
> 0	6 (12.6)
Self-rated health status pre-COVID-19	8.6 ± 1.2
Post-exertional malaise	
No	7 (14.9)
Yes	40 (85.1)
Cognitive impairment	
No	20 (42.6)
Yes	27 (57.4)
Pre-existing diseases	
None	14 (2.3)
Thyroid disease	11 (23.4)
Cardio-vascular disease	6 (12.8)
Arterial hypertension	6 (12.8)
Pre-existing mental disorders	6 (12.8)
Chronic inflammatory skin disease	5 (10.6)
Gastrointestinal disease	4 (8.5)
Allergies	4 (8.5)
Asthma	3 (6.4)
Migraine	3 (6.4)
Rheumatism	2 (4.3)
Obesity	2 (4.3)
Diabetes	1 (2.1)

^*^Data are presented as mean ± SD (standard deviation), median and IQR (quartile 1–quartile 3), or absolute and relative frequencies (%)

### Effect of intervention on outcome measures

**Fig. 3:**
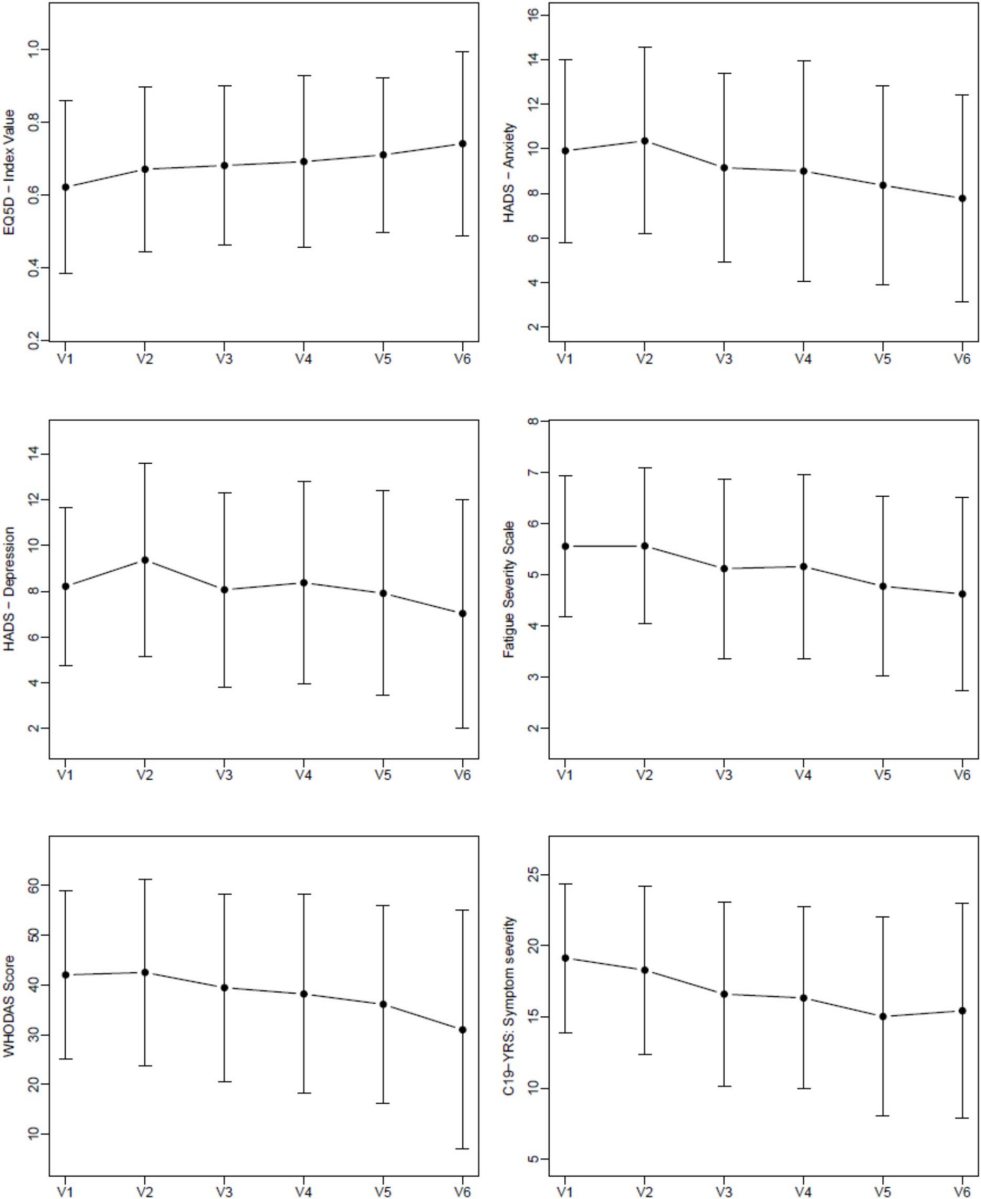
Mean values of health-related quality of life, psychological burden, fatigue, functional disability, and symptom severity over time (V1–V6). Points represent means at each time-point; vertical bars indicate ± standard deviation. Health-related quality of life was assessed using the EQ-5D index value, anxiety and depression using the Hospital Anxiety and Depression Scale (HADS), fatigue using the Fatigue Severity Scale (FSS), functional disability using the WHODAS-12 score, and overall symptom severity using the modified COVID-19 Yorkshire Rehabilitation Screening test

Descriptive statistics and corresponding plots of the outcome measures from all study visits are shown in Table 2, Fig. 3, and Supplementary Fig. 1. Numbers of available outcome data per study visit are shown in Supplementary Table 2. The results of the longitudinal mixed model are shown in Table 3.

**Table 2:** Descriptive statistics of the outcomes over the course of the study

	Visit 1	Visit 2	Visit 3	Visit 4	Visit 5	Visit 6
EQ-5D-5L	0.62 ± 0.24	0.67 ± 0.23	0.68 ± 0.22	0.69 ± 0.24	0.71 ± 0.21	0.74 ± 0.25
Fatigue-severity-scale-7	5.6 ± 1.4	5.6 ± 1.5	5.1 ± 1.8	5.2 ± 1.8	4.8 ± 1.8	4.6 ± 1.9
HADS						
Anxiety	10 (7.5–12)	11 (7–13.5)	8.5 (7–12)	9 (6–12)	8 (5–12)	7.5 (4–11)
Depression	8 (6–10)	9 (6.5–12)	8 (6–10)	7 (5–11)	7.5 (5–10)	7 (3–9)
WHODAS-12 score, %	42.0 ± 16.8	42.5 ± 18.7	39.5 ± 18.9	38.2 ± 20.0	36.1 ± 19.9	31.0 ± 24.0
C19-YRS						
Symptom severity	19.1 ± 5.2	18.3 ± 5.9	16.6 ± 6.4	16.3 ± 6.4	15.0 ± 7.0	15.4 ± 7.5
Functional disability	6.7 ± 3.4	6.8 ± 3.2	5.8 ± 3.5	5.6 ± 3.4	5.2 ± 3.6	4.8 ± 4.1
Health score	4.2 ± 1.7	3.7 ± 1.9	4.7 ± 2.1	4.7 ± 2.1	5.1 ± 2.2	5.4 ± 2.4
Mod. Medical Research Council Dyspnea Scale	1 (0–2)	1 (0–2)	1 (0–2)	1 (0–2)	0 (0–1)	1 (0–2)
MoCA						
MoCA blind	19.5 ± 1.7	19.8 ± 1.8	20.3 ± 1.5	19.9 ± 1.7	19.9 ± 1.5	19.4 ± 1.5
MoCA	26.6 ± 2.3	27.3 ± 2.0	27.8 ± 1.7	27.7 ± 1.8	N/A	N/A
Handgrip strength max. [kg]	34.5 ± 13.2	33.7 ± 13.2	34.0 ± 15.0	35.1 ± 13.7	N/A	N/A
Two-minute walk test [m]	130.4 ± 24.6	127.7 ± 24.4	126.3 ± 26.0	136.4 ± 22.8	N/A	N/A
Mini-BESTest	23.5 ± 4.1	24.4 ± 3.9	24.5 ± 4.0	25.1 ± 3.2	N/A	N/A

Data are presented as n (%), mean ± SD or median (quartile 1–quartile 3)

*C19-YRS * modified COVID-19 Yorkshire Rehabilitation Screening, *HADS*  hospital anxiety and depression scale, *EQ-5D-5l *EuroQol—5 dimensions—5 level, *MoCA* montreal cognitive assessment, *Mini-BESTest* mini-balance evaluation systems test, *WHODAS-12 * world health organization disability assessment schedule 2.0—12 items

**Table 3:** Results of the longitudinal mixed models

	Change within…				
	Intervention period (V2–V3)	Two weeks post-intervention (V3–V4)	Two months post-intervention (V4–V5)	Six months post-intervention (V5–V6)	ICC
EQ-5D-5L—Index value	0.01 (0.720)	0.01 (0.794)	0.02 (0.391)	0.04 (0.185)	0.553
Fatigue-severity-scale-7	**–0.44 (0.017)**	0.05 (0.790)	–0.36 (0.059)	–0.19 (0.355)	0.643
HADS					
Anxiety	**–1.20 (0.008)**	–0.10 (0.831)	–0.64 (0.169)	–0.61 (0.205)	0.741
Depression	**–1.36 (0.002)**	0.39 (0.370)	–0.36 (0.416)	**–0.95 (0.037)**	0.751
WHODAS-12 score, %	–3.06 (0.091)	–0.99 (0.586)	–2.34 (0.211)	**–5.87 (0.003)**	0.756
C19-YRS					
Symptom severity	**–1.68 (0.018)**	–0.21 (0.773)	–1.29 (0.074)	0.02 (0.979)	0.678
Functional disability	**–0.98 (0.005)**	–0.17 (0.626)	–0.43 (0.227)	–0.63 (0.092)	0.637
Health score	**0.91 (0.004)**	0.07 (0.832)	0.29 (0.366)	0.48 (0.153)	0.377
Mod. Medical Research Council Dyspnea Scale	**–0.30 (0.012)**	0.05 (0.706)	–0.22 (0.072)	**0.26 (0.047)**	0.558
MoCA blind	0.52 (0.063)	–0.43 (0.132)	–0.07 (0.812)	–0.49 (0.094)	0.247
MoCA	0.46 (0.162)	–0.15 (0.664)			0.279
Handgrip strength max. [kg]	0.45 (0.595)	–0.05 (0.958)			0.703
Two-minute walk test [m]	–0.94 (0.709)	**7.21 (0.007)**			0.758
Mini-BESTest	0.23 (0.398)	0.02 (0.994)			0.882

Coefficients are presented as the change in the respective period compared to the preceding period and are corrected for age, sex, cognitive impairment, post-exertional malaise and health status pre-COVID-19. P-values are in brackets and represent if the outcome level was significantly different from the change in the preceding period (e.g., the change in the intervention period is compared to the change in the control period and the 2 weeks post-intervention to the intervention period)

*C19-YRS* modified COVID-19 Yorkshire Rehabilitation Screening, *HADS *hospital anxiety and depression scale, *EQ-5D-5L *EuroQol—5 dimensions—5 level, *MoCA* montreal cognitive assessment, *Mini-BESTest* mini-balance evaluation systems test, *WHODAS-12*  world health organization disability assessment schedule 2.0—12 items

Significant values are in bold

No significant improvements could be shown for HRQoL, WHODAS-12, and all performance measures (MoCA, handgrip strength, walking, and balance) during the intervention period (*p* > 0.05) compared to the preceding control period (Table 3). Significant decreases in fatigue, anxiety, depression, symptom severity, functional disability, and dyspnea, and a better subjective health score were observed during the intervention period compared to the change in the control period. At the start of the intervention, 83% reported substantial fatigue, 70% from anxiety, and 66% from depression. After the intervention, percentages decreased to 74% (fatigue), 61% (anxiety), and 54% (depression).

Descriptive statistics, corresponding plots and results of the longitudinal mixed model of the DePaul Symptom Questionnaire are displayed in Supplementary Table 3 and Supplementary Fig. 2. The reduction of severity during the intervention period of the items 3 (minimum exercise makes you physically tired), 7 (problems remembering things), 10 (feeling unsteady on your feet), and 11 (cold limbs) was significantly different from the change in the control period.

### Effect of risk factors on outcome measures

Supplementary Table 4 presents the results of the longitudinal mixed model including the adjustments for confounders and interactions. Age was significantly negatively associated with fatigue (−0.03, [95% CI −0.05; 0]; *p* = 0.024), and grip strength (−0.30 [95% CI −0.42; −0.17]; *p* < 0.001). Female sex was negatively associated with grip strength (−21.87 [95% CI −25.73; −18.02]; *p* < 0.001), disability according to the WHODAS-12 (12.05 [95% CI 2.80; 21.30]; *p* = 0.017), functional disability (2.28 [95% CI 0.79; 3.78]; *p* = 0.004), and the health score (−1.04 [95% CI −1.83; −0.26]; *p* = 0.012). Cognitive impairment at V1 was not significantly associated with any of the outcomes. PEM was significantly associated with higher functional disability (2.23 [95% CI 0.14; 4.33]; *p* = 0.041), but with less cognitive impairment according to the MoCA (1.76 [95% CI 0.71; 2.81], *p* = 0.002). A higher subjective health score before COVID-19 was significantly associated with less fatigue, anxiety, depression, symptom severity, functional disability, and dyspnea (Supplementary Table 4).

### Interaction of confounding variables with time

**Fig. 4:**
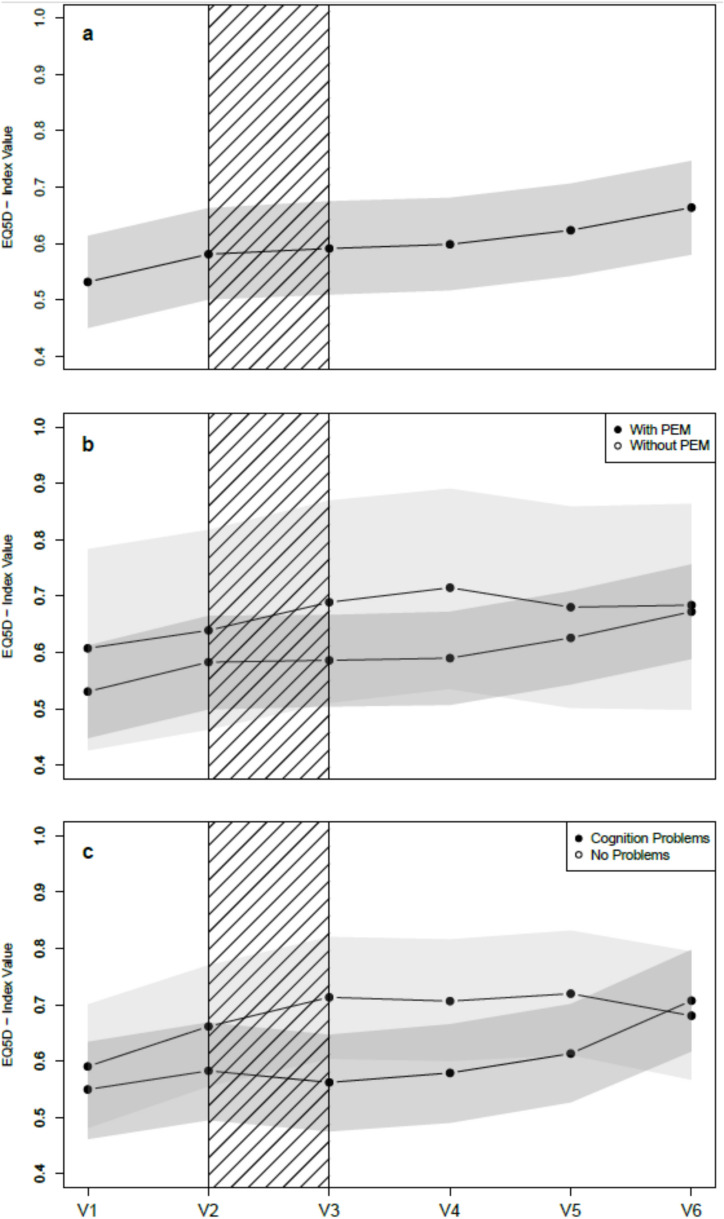
Predicted EQ-5D-5L index values over time for a 49 year-old female participant based on the linear mixed-effects model. Solid lines denote predicted means; grey bands show point-wise bootstrap 95% confidence intervals. The hatched area indicates the 2 week intervention period. Panel **a** shows the trajectories for the whole sample; **b** for patients with and without post-exertional malaise (PEM); and **c** for patients with and without cognitive problems

A significant different progress of health-related quality of life over time was found for individuals with and without cognitive impairment (Fig. 4; *p* = 0.038). The progress in MoCA blind differed for patients with and without PEM (*p* = 0.046; Supplementary Table 4). No further significant interactions were found for PEM or cognitive impairment over time (Supplementary Table 4).

### Effect on steps per day and resting heart rate

Steps per day and resting heart rate were not available for all patients due to technical issues or lack of compliance. Resting heart rate (beats per minute) did not change significantly over time and was on average (± standard deviation) 69.2 ± 8.8 in the 2 weeks before therapy (available measurements *n* = 31), 68.1 ± 9.4 during the 2 weeks of on-site intervention (*n* = 35), 66.6 ± 8.4 in the 2 weeks after the therapy intervention (*n* = 33), and 66.8 ± 8.0 in the 2 weeks around the first follow-up measurement V5 (*n* = 24). Mean number of steps per day was 8358 ± 4313 in the 2 weeks before therapy (*n* = 34), 9551 ± 3414 during the on-site therapy (*n* = 37), 8335 ± 4023 in the 2 weeks after the therapy intervention (*n* = 34), and 7849 ± 3358 in the 2 weeks around V5 (*n* = 28). Participants conducted on average 1,000 more steps per day during the intervention compared to the control period (*p* = 0.024).

### Long-term change

At the follow-up visits V5 and V6, health-related quality of life and all other outcome measures have improved in comparison to the values measured at V1 (except for MoCA blind at V6; Table 2). Regarding the DePaul Symptom Questionnaire, most items also have improved over time compared to the values at V1 (Supplementary Table 3).

### Continuous app-supported home training

Nine participants (19.1%) did not use or try the digital intervention at all. Six more persons (12.8%) used the app only once or twice and had an overall participation of less than 2.0%. Regarding the remaining persons, overall participation in the digital intervention was 20.9 ± 19.4%. Participation in the category exercises was higher (25.9 ± 24.5%) compared to the category education (14.2 ± 20.8%). The level of overall participation exhibited its peak during the initial week (37.5 ± 23.4%) and progressively declined thereafter, reaching its nadir during the eighth and final week with a participation rate of 8.6 ± 20.0%. A total of 31 participants rated the usefulness and helpfulness of the app. Fourteen (45.2%) found it useful, eleven (35.5%) expressed neutrality, and six (19.4%) indicated that they did not perceive the app useful or helpful. Main reasons for not regularly using the app were not enough time in daily life and the preference of other exercises.

### Satisfaction with the on-site therapy intervention

The most popular therapies among the participants were peat bath (66.0%), massage (53.3%), and breathing therapy (48.9%). The therapies that were least liked were balance and cognitive training guided by a multifunctional rehabilitation device (34.0%), progressive muscle relaxation (29.8%), and autogenic training (25.5%). Most frequently, participants found the on-site therapy intervention beneficial for respiratory difficulties (46.8%) and cognitive function (25.5%). In contrast, participants found that the intervention to be least effective for fatigue, both physically (21.3%) and mentally (19.1%), as well as for pain (17.0%). In addition, 56.5% considered the number of therapies to be too high, 34.8% found it appropriate, and only 8.7% felt it was insufficient. In general, patients were quite satisfied with the on-site therapy (Table 4).

**Table 4:** Satisfaction with the on-site therapy

Questions	Median (Q1–Q3)	Mean ± SD
1. My needs were met by the therapy	4 (3–4)	3.6 ± 0.9
2. I am satisfied with the therapy	4 (3–4)	3.9 ± 0.9
3. I would recommend the therapy to a friend with a similar issue	4 (4–5)	4.1 ± 0.9
4. The therapy helps me cope with my COVID long-term effects	4 (3–4)	3.7 ± 0.8
5. The information provided is very helpful to me	4 (4–5)	4.2 ± 0.8
6. I felt comfortable in the program	5 (3–5)	4.1 ± 1.1
7. I have learned helpful strategies for dealing with the COVID long-term effects	4 (4–5)	3.9 ± 0.9
8. I am satisfied with the quality of the therapy I received	4 (4–5)	4.1 ± 0.9
9. I am now able to effectively manage my problems	4 (3–4)	3.7 ± 0.9

Each item on the questionnaire was rated on a Likert-scale (1: I completely disagree; 2: I disagree; 3: neutral; 4: I agree, 5: I completely agree)

## Discussion

In this study, we aimed to evaluate a multidisciplinary therapy program targeted at neurological symptoms of persons with post-COVID-19 condition. After the completion of the therapy program, we observed statistically significant improvements in several patient-reported outcomes, including fatigue, anxiety, depression, dyspnea, all subcategories of the C19-YRS (symptom severity, functional disability, health score), and parts of the DePaul Symptom Questionnaire. However, no significant improvements were observed in HRQoL, WHODAS-12, or performance measures.

### Effect of the program on neurological symptoms

We found statistically significant improvements in fatigue after the completion of the therapy intervention. This result is in line with a recent study, where a significant reduction of fatigue was shown after a 6-week rehabilitation program, consisting of aerobic exercises, resistance training, and educational sessions twice a week [50]. Furthermore, a significant decrease of fatigue levels was also demonstrated in a meta-analysis investigating the effect of physical exercise-based rehabilitation on post-COVID-19 condition [51] and a meta-analysis about rehabilitation interventions for old adults with post-COVID-19 condition [52].

We did not observe significant improvements in cognitive function. In contrast, other studies have reported significant cognitive gains after physical exercise-based rehabilitation programs [51] or multidisciplinary rehabilitation approaches including neuropsychological treatment [53]. The relatively high mean MoCA score at baseline in our sample suggests low cognitive impairment, which may partly explain the absence of measurable effects [54]. Notably, persistent deficits in attention and working memory have been reported in a 5 week inpatient rehabilitation setting, even when using more comprehensive cognitive test batteries [55]. Thus, further research is needed to better understand and improve cognitive rehabilitation in individuals affected by post-COVID-19 condition.

The neuropsychological symptoms anxiety and depression both improved significantly after our therapy intervention. Decreases in depressive symptoms were previously observed after rehabilitation [51, 55]. Evidence for the effect of anxiety remains inconclusive, as some studies suggest a potential benefit of rehabilitation for anxiety symptoms [52, 56, 57], while others show no significant impact [51].

### Rehabilitation of the post-COVID-19 condition

In meta-analyses about the effects of rehabilitation in persons with post-COVID-19 condition, beneficial effects on health-related quality of life, dyspnea, and the 6 min walking test were observed (besides mental health and fatigue, as discussed previously) [51, 52]. An 8 week multicomponent exercise program including resistance and endurance sessions along with or without inspiratory muscle training showed significant improvements in dyspnea, fatigue, and depression, similar to our findings. Furthermore, significant improvements in health-related quality of life and muscle strength were observed [58]. Other studies on rehabilitation programs similar to ours have reported comparable improvements in cardiopulmonary health, fatigue, functional capacity, and certain aspects of health-related quality of life [59–62]. An 8-week outpatient rehabilitation program, for example, demonstrated effectiveness in reducing cognitive and emotional symptoms in individuals with post-COVID-19 condition [63]. Additional studies have shown promising results regarding neurological post-COVID-19 symptoms, including improvements in neurocognitive functions, sleep quality, fatigue, psychiatric symptoms, and health-related quality of life [64–68]. Although these studies illustrate alternative multidisciplinary therapeutic approaches, direct comparisons with our findings are limited due to differences in intervention duration, therapeutic modalities, and outcome assessment methods.

To sum up, there is emerging evidence that rehabilitation therapies can reduce (neurological) symptoms of the post-COVID-19 condition. However, knowledge about the role of neurorehabilitation and effective therapies is still scarce. Several suggested therapies (e.g., energy management education, heart rate monitoring) still need to be evaluated [28]. Rehabilitative therapies may serve as a crucial adjunct to drug therapy in the treatment of post-COVID-19 conditions, especially considering that no definitive drug treatments have yet been proven effective, and several clinical trials are still ongoing [69].

Although post-COVID-19 condition represents a distinct clinical entity, the design of our multidisciplinary rehabilitation program was informed by established principles of neurorehabilitation derived from large randomized controlled trials in other neurological disorders, such as Parkinson’s disease, which already have demonstrated the benefits of structured, exercise-based and multimodal rehabilitation approaches [70–73].

### Long-term trajectories of the post-COVID-19 condition

Although our study was not designed to evaluate long-term effects, the 6-month follow-up showed improvements in nearly all neurological outcome measures. This pattern is consistent with a large cohort, in which the majority of patients (91%) exhibited a gradual decline in symptom burden over 2 years [16]. Improvements in pulmonary function and shortness of breath were reported up to 2 years after infection [74–76]. Furthermore, decreases in cognitive symptoms, depressive symptoms, and sleeping disorder were reported. [76, 77] Findings for fatigue are heterogeneous, with reports of improvement [76], stability, or even worsening over time [57, 75, 78]. Notably, no worsening trajectory was observed in our cohort, which may reflect natural long-term recovery. Although a potential stabilizing effect of the combined on-site and digital intervention cannot be excluded, causal conclusions cannot be drawn due to the lack of a control group.

### Post-exertional malaise

Some of the symptoms of the post-COVID-19 condition overlap with the myalgic encephalomyelitis/chronic fatigue syndrome (ME/CFS) [69, 79]. One main symptom of ME/CFS is PEM, the worsening of symptoms after activity [30]. The main coping strategy is energy management/pacing which was associated with better outcomes and fewer post-exertional symptom exacerbations in patients with post-COVID-19 condition [80, 81], although study results are heterogeneous and lack high quality [30]. Physical exercises and regular rehabilitation were frequently proposed in patients with post-COVID-19 condition, and it was shown to be beneficial in several cases [82]. However, many individuals with post-COVID-19 condition meet the criteria for ME/CFS [83], and it was stated that these patients should not engage in conventional exercise programs, as this could even be harmful by triggering relapses/PEM [43–45, 82]. We did not implement a pacing protocol, but participants were taught about the concept, and pacing was considered in each unit of the program. To control for PEM in the analyses, we classified patients with PEM and included the condition as a covariate. No significant interactions with PEM were found.

### Limitations

We acknowledge several limitations in our study. Primarily, since our objective was to develop an intervention, the absence of a control group prevents us from drawing definitive conclusions about the efficacy of our therapy program. The results should be interpreted as exploratory and intended to inform and guide the development of future randomized controlled trials. However, by comparing changes in outcomes during the pre-study period with those observed during the intervention period within the same study sample, we could mitigate the impact of biases such as selection bias, i.e., by selecting an inappropriate control group or confounding by natural disease progression. In addition, incomplete participation in the on-site therapy program further complicates our analysis. Data about therapy utilization were only collected in a subset of participants; thus, we were not able to incorporate this information in our analyses. A further limitation is the lack of a sample size calculation, due to the exploratory character of the study. Consequently, the study was not powered to detect small-to-moderate effects in patient-reported outcomes. Both significant and non-significant findings therefore need to be interpreted with caution.

Another limitation is the comparatively short duration of the in-person therapy intervention. As mentioned in the systematic review by Zheng et al. [51], rehabilitation programs for post-COVID-19 conditions typically extend over 6 weeks or longer. Differences in intervention duration may partly explain discrepancies between our findings and those reported in the literature and should be considered when interpreting the results.

We intended to prolong the rehabilitation period through an 8 week app-supported home training program, but low engagement within this program represents another limitation: one-third of participants did not use the app, while two-thirds had a median usage of 20%. Barriers include live and work circumstances, alternative therapy preferences, and digital difficulties. Despite reminders from the study personnel, enhancing usage rates would have necessitated additional efforts. Thus, we cannot draw definite conclusions on the app’s benefits.

Positive group dynamics can significantly contribute to a successful outcome, and several participants mentioned the positive influence of other group members. However, group dynamics varied noticeably among the ten study circles, and unfortunately, we did not measure this aspect in our study.

Strengths of the study were its longitudinal design, the high number of different outcome measures (including several patient-reported outcome measures), and the long follow-up with a good response rate.

## Conclusion

We developed and evaluated a multidisciplinary neurological therapy program for individuals with post-COVID-19 condition. Compared to the preceding control period, improvements were observed in symptoms such as fatigue, anxiety, depression, and dyspnea, as well as improvements in symptom severity and functional disability during the 2 week intervention. Changes in outcomes over time did not differ for patients with PEM or cognitive impairment. Given the exploratory design, lack of a control group, absence of a formal sample size calculation, and the short intervention duration, these findings, although promising, should be interpreted cautiously. Nevertheless, the results suggest that multidisciplinary rehabilitation approaches may have the potential to address neurological and non-neurological symptoms of post-COVID-19 condition.

Further high-quality research, including randomized controlled trials, is essential to confirm these findings and to better understand the effectiveness of multidisciplinary therapies in treating post-COVID-19 conditions.

## Supplementary Information

Below is the link to the electronic supplementary material.

Supplementary file1 (DOCX 289 KB)

## Data Availability

Raw data of ther study can be provided in case of a reasonable request.
