# Supplementary material for: Evaluation of a multidisciplinary neurological rehabilitation program for the post-COVID-19 condition
Source: J Neurol. 2026 Feb 22;273(2):159. doi: 10.1007/s00415-026-13693-5 (PMC12926238; doi:10.1007/s00415-026-13693-5)
Supplement: Supplementary file 1 — Supplementary file1 (DOCX 289 KB) [file 415_2026_13693_MOESM1_ESM.docx]

**A multidisciplinary neurological rehabilitation program
for the post COVID-19 condition**

Marion Egger^1*^, Ralf Strobl^2,3*^, Lena Vogelgesang^1^, Judith Reitelbach^1^,
Eva Grill^2,3^, Klaus Jahn^1,3 §^

^1^Schoen Clinic Bad Aibling, Department of Neurology, Research Group, Bad Aibling, Germany;
^2^Institute of Medical Data Processing, Biometrics and Epidemiology, Faculty of Medicine, LMU Munich, Munich Germany
^3^German Center for Vertigo and Balance Disorders, LMU University Hospital, LMU Munich, Germany

* Shared first authorship

§ Corresponding author

Table of Contents

[Supplementary Table 1 Overview therapy interventions 2](#_Toc219454006)

[Supplementary Information 1: Example of the two-week therapy plan 3](#_Toc219454007)

[Supplementary Information 2: Digital Intervention 5](#_Toc219454008)

[Supplementary Information 3: Detailed description of secondary outcome measures 10](#_Toc219454009)

[Supplementary Table 2 Number of available outcome data per study visit 13](#_Toc219454010)

[Supplementary Figure 1 14](#_Toc219454011)

[Supplementary Figure 2 15](#_Toc219454012)

[Supplementary Table 3 Descriptive summary of the DePaul Symptom Questionnaire (short form) and results of the longitudinal mixed models. 16](#_Toc219454013)

[Supplementary Table 4 Effect of the risk factors age, sex, post-exertional malaise (PEM), cognition, subjective health score and interaction terms of time with PEM and cognition, respectively 18](#_Toc219454014)

[References 20](#_Toc219454015)

| Supplementary Table 1 Overview therapy interventions | | |
| --- | --- | --- |
| **Type^1^** | **Time per session** | **Number within 10 working days** |
| Nordic walking ^2^ | 60min | 4 |
| Peat bath and massage ^3^ | 120 min (including resting time) | 4 |
| Qi Gong | 60min | 2 |
| Progressive muscle relaxation | 60min | 2 |
| Autogenic training | 60min | 2 |
| Balance training (posture & gait) ^4^ | 30min | 12 |
| Breathing therapy^5^ | 30min | 4 |
| Cognitive computer training^6^ | 30min | 3-4 |
| Balance and cognitive training with Pablo® (tyromotion, Austria) ^7^ | 45min | 4 |
| Neuropsychologist consultation | 60min | 2 |
| Neurologist consultation | 15min | 2 |
| Psychological information ^8^ | 60min | 1 |
| Resistance training with strength training equipment^9^ | 60min | 4 |
| Note: An haulage service transported the participants to each therapy session and the joint lunch in Bad Aibling. In the afternoon, all therapies took place at the Schoen Clinic.  ^1^ Most therapy sessions were conducted in group settings except for the peat bath and massage, cognitive computer training, Pablo training and the neurologist and neuropsychologist consultation.  ^2^ Individually adapted distance and pace, including a warm-up and stretching as cool-down.  ^3^ Patients could choose from either a full-body bath, a partial bath, or just a mud pack, depending on their tolerance and preference.  ^4^  Gait variations, stepper training, endurance training (ring hockey), table tennis, badminton, exercises with the Pezzi ball or balance pad, training with the Wii, coordination training with Indiaca paddles and Koosh balls, break management (active through stretching exercises or passive)  ^5^ Education on physiological beathing patterns (airways, nasal beathing, diaphragmatic beathing, rhythm), education on lip beathing, trunk mobilization exercises, breathing exercises (box breathing, interval breathing with focus on exhalation), information on accessory breathing muscles, breath-enhancing positions, breath away skin folds etc.  ^6^ The exercises were selected based on a prior Attention Assessment Test Battery. The level of difficulty and type of exercise were tailored to the patient. The sessions primarily focused on exercises for divided attention, reaction speed, and sustained attention; additionally, memory exercises were conducted. Primarily, exercises from Cogpack, Rehacom, and Freshminder were utilized, as well as Rigling programs.  ^7^ The Pablo device enhances rehabilitation through interactive therapy sessions designed for motor function and coordination improvement. Patients engage in various interactive games that require upper limb movements, promoting strength and range of motion while ensuring motivation. The device provides real-time visual and auditory feedback. The therapy can take place in sitting or standing position. Additionally, the therapy employs a multisensory approach, combining visual and tactile stimuli to increase engagement, making rehabilitation effective and enjoyable for patients.  ^8^ Self-care, sleep hygiene, pacing (i.e. prevention of post exertional malaise and crashes)  ^9^ Type and intensity were individually adapted to the patients. Machines like leg press, cable pulley systems and ergometer (for warm-up) were used. | | |

# Supplementary Information 1: Example of the two-week therapy plan

**Therapy plan - week 1**

|  | **Monday** | **Tuesday** | **Wednesday** | **Thursday** | **Friday** |
| --- | --- | --- | --- | --- | --- |
| 08.30 |  | Nordic Walking |  | Nordic Walking |  |
| 08.45 |  |  |  |  |  |
| **09.00 am** | Study visit 2 |  | Resistance training |  | Resistance training |
| 09.15 |  |  |  |  |  |
| 09.30 |  | Moor bath and massage |  | Moor bath and massage |  |
| 09.45 |  |  |  |  |  |
| **10.00** |  |  |  |  |  |
| 10.15 |  |  |  |  |  |
| 10.30 |  |  |  |  |  |
| 10.45 |  |  |  |  |  |
| **11.00** | Welcome, introduction, information |  | Qigong |  | Qigong |
| 11.15 |  |  |  |  |  |
| 11.30 |  |  |  |  |  |
| 11.45 |  |  |  |  |  |
| **12.00** | **Joint lunch** | | | | |
| 12.15 |  |  |  |  |  |
| 12.30 |  |  |  |  |  |
| 12.45 |  |  |  |  |  |
| **01.00 pm** | Balance (gait) | Balance (gait) | Breathing therapy | Breathing therapy | Balance (gait) |
| 01.15 |  |  |  |  |  |
| 01.30 |  |  |  |  |  |
| 01.45 | Pablo balance training |  | Pablo balance training |  |  |
| **02.00** |  |  |  |  |  |
| 02.15 |  |  |  |  |  |
| 02.30 | **Physician consultation** | Cognitive training |  | Cognitive training | Cognitive training |
| 02.45 |  |  |  |  |  |
| **03.00** | Neuro-psychologist consultation |  | Cognitive training |  |  |
| 03.15 |  |  |  |  |  |
| 03.30 |  | Balance (posture) |  | Balance (posture) | Balance (posture) |
| 03.45 |  |  |  |  |  |
| **04.00** |  |  | Autogenous training |  |  |
| 04.15 |  |  |  |  |  |
| 04.30 |  |  |  |  |  |
| 04.45 |  |  |  |  |  |
| **05.00** |  |  |  |  |  |
| 05.15 |  |  |  |  |  |
| 05.30 |  |  |  |  |  |
| 05.45 |  |  |  |  |  |

**Therapy plan - week 2**

|  | **Monday** | **Tuesday** | **Wednesday** | **Thursday** | **Friday** |
| --- | --- | --- | --- | --- | --- |
| 08.30 |  | Nordic Walking |  | Nordic Walking |  |
| 08.45 |  |  |  |  |  |
| **09.00 am** | Resistance training |  | Resistance training |  | Study visit 3 |
| 09.15 |  |  |  |  |  |
| 09.30 |  | Moor bath and massage |  | Moor bath and massage |  |
| 09.45 |  |  |  |  |  |
| **10.00** |  |  |  |  |  |
| 10.15 |  |  |  |  |  |
| 10.30 | Progressive muscle relaxation |  | Progressive muscle relaxation |  | Caspar App |
| 10.45 |  |  |  |  | Feedback round, closing |
| **11.00** |  |  |  |  |  |
| 11.15 |  |  |  |  |  |
| 11.30 |  |  |  |  |  |
| 11.45 |  |  |  |  |  |
| **12.00** | **Joint lunch** | | | | |
| 12.15 |  |  |  |  |  |
| 12.30 |  |  |  |  |  |
| 12.45 |  |  |  |  |  |
| **01.00 pm** | Balance (gait) | Balance (gait) | Breathing therapy | Breathing therapy | Balance (gait) |
| 01.15 |  |  |  |  |  |
| 01.30 |  |  |  |  |  |
| 01.45 | Pablo balance training |  | Pablo balance training |  |  |
| **02.00** |  |  |  |  |  |
| 02.15 |  |  |  |  |  |
| 02.30 |  | Cognitive training |  | Cognitive training | Cognitive training |
| 02.45 |  |  |  |  |  |
| **03.00** | Neuro-psychologist consultation |  | Cognitive training |  |  |
| 03.15 |  |  |  |  |  |
| 03.30 |  | Balance (posture) |  | Balance (posture) | Balance (posture) |
| 03.45 |  |  |  |  |  |
| **04.00** |  | Psychological information | Autogenous training | **Physician consultation** |  |
| 04.15 |  |  |  |  |  |
| 04.30 |  |  |  |  |  |
| 04.45 |  |  |  |  |  |
| **05.00** |  |  |  |  |  |
| 05.15 |  |  |  |  |  |
| 05.30 |  |  |  |  |  |
| 05.45 |  |  |  |  |  |

# Supplementary Information 2: Digital Intervention

**Exercise** **videos**: E.g. balance and resistance training, stretching, breathing, and relaxation exercises (progressive muscle relaxation, autogenous training)

**Educational videos**: E.g. stress management, healthy diet, mindfulness meditation, anxiety disorders, sleep hygiene.

Exercise and educational video duration per day was 30-60 minutes. The therapy schedule (see Supplement) was individually adapted upon request of the participants (e.g. if one exercise was painful or if more resistance exercises were desired).

The first two weeks of the CASPAR training program are presented as illustrative examples in the following.

**Caspar Therapy Week 1**

| **Day 1 (62 min)** | **Day 2 (52 min)** | **Day 3 (61 min)** | **Day 4 (49 min)** | **Day 5 (54 min)** | **Day 6 (48 min)** | **Day 7 (75 min)** |
| --- | --- | --- | --- | --- | --- | --- |
| #901 Guided imagery – The white rose | Autogenic training – Warmth exercise | #900 Guided imagery – Walk in the park | Progressive muscle relaxation (Jacobson) | #899 Guided imagery – Walk in the forest | Autogenic training – Heaviness exercise | Stress and stress management – Part 1 |
| #836 Side tapping with arm movement in standing – warm-up / endurance | Mindfulness: 3-2-1 strategy | #836 Side tapping with arm movement in standing – warm-up / endurance | #357 Stress management – Focusing on the positive | #836 Side tapping with arm movement in standing – warm-up / endurance | #836 Side tapping with arm movement in standing – warm-up / endurance | Experiencing the present moment – Basic principles of mindfulness meditation |
| #770 Small arm circles – warm-up / endurance | #836 Side tapping with arm movement in standing – warm-up / endurance | #770 Small arm circles – warm-up / endurance | #836 Side tapping with arm movement in standing – warm-up / endurance | #770 Small arm circles – warm-up / endurance | #770 Small arm circles – warm-up / endurance | #836 Side tapping with arm movement in standing – warm-up / endurance |
| #922 Sternal breathing in sitting | #770 Small arm circles – warm-up / endurance | #922 Sternal breathing in sitting | #770 Small arm circles – warm-up / endurance | #922 Sternal breathing in sitting | #922 Sternal breathing in sitting | #770 Small arm circles – warm-up / endurance |
| #921 Lateral breathing in sitting | #922 Sternal breathing in sitting | #921 Lateral breathing in sitting | #922 Sternal breathing in sitting | #921 Lateral breathing in sitting | #921 Lateral breathing in sitting | #922 Sternal breathing in sitting |
| #066 Strengthening the knee extensors by sit-to-stand | #921 Lateral breathing in sitting | #066 Strengthening the knee extensors by sit-to-stand | #921 Lateral breathing in sitting | #066 Strengthening the knee extensors by sit-to-stand | #066 Strengthening the knee extensors by sit-to-stand | #921 Lateral breathing in sitting |
| #299 Balancing on tiptoes on a line | #066 Strengthening the knee extensors by sit-to-stand | #299 Balancing on tiptoes on a line | #066 Strengthening the knee extensors by sit-to-stand | #299 Balancing on tiptoes on a line | #299 Balancing on tiptoes on a line | #066 Strengthening the knee extensors by sit-to-stand |
| #382 Cervical spine stretching | #299 Balancing on tiptoes on a line | #382 Cervical spine stretching | #299 Balancing on tiptoes on a line | #382 Cervical spine stretching | #382 Cervical spine stretching | #299 Balancing on tiptoes on a line |
| #366 Cat–cow in quadruped position – spinal mobilization | #382 Cervical spine stretching | #366 Cat–cow in quadruped position – spinal mobilization | #382 Cervical spine stretching | #366 Cat–cow in quadruped position – spinal mobilization | #366 Cat–cow in quadruped position – spinal mobilization | #382 Cervical spine stretching |
|  | #366 Cat–cow in quadruped position – spinal mobilization |  | #366 Cat–cow in quadruped position – spinal mobilization |  |  | #366 Cat–cow in quadruped position – spinal mobilization |

**Caspar Therapy Week 2**

| **Day 8 (49 min)** | **Day 9 (44 min)** | **Day 10 (50 min)** | **Day 11 (37 min)** | **Day 12 (53 min)** | **Day 13 (38 min)** | **Day 14 (64 min)** |
| --- | --- | --- | --- | --- | --- | --- |
| #898 Guided imagery – Walk through the autumn forest | Autogenic training – Warmth exercise | #353 Stress management – Health risks of stress | Progressive muscle relaxation (Jacobson) | #896 Guided imagery – Bathing joy | Autogenic training – Full body exercise | Healthy nutrition – Part 1 |
| #771 Hip circles – warm-up / endurance | Mindfulness: mindful eating | #897 Guided imagery – Returning to the path | #771 Hip circles – warm-up / endurance | #771 Hip circles – warm-up / endurance | #771 Hip circles – warm-up / endurance | Progressive muscle relaxation (Jacobson) |
| #781 Standing core strengthening | #771 Hip circles – warm-up / endurance | #771 Hip circles – warm-up / endurance | #781 Standing core strengthening | #781 Standing core strengthening | #781 Standing core strengthening | Stress and stress management – Part 2 |
| #825 Upright posture of the spine – mobilization / strengthening | #781 Standing core strengthening | #781 Standing core strengthening | #825 Upright posture of the spine – mobilization / strengthening | #825 Upright posture of the spine – mobilization / strengthening | #825 Upright posture of the spine – mobilization / strengthening | #771 Hip circles – warm-up / endurance |
| #296 Standing on tiptoes with eyes closed | #825 Upright posture of the spine – mobilization / strengthening | #825 Upright posture of the spine – mobilization / strengthening | #296 Standing on tiptoes with eyes closed | #296 Standing on tiptoes with eyes closed | #296 Standing on tiptoes with eyes closed | #781 Standing core strengthening |
| #924 Seated spinal flexion with leg lift – breathing / mobilization | #296 Standing on tiptoes with eyes closed | #296 Standing on tiptoes with eyes closed | #924 Seated spinal flexion with leg lift – breathing / mobilization | #924 Seated spinal flexion with leg lift – breathing / mobilization | #924 Seated spinal flexion with leg lift – breathing / mobilization | #825 Upright posture of the spine – mobilization / strengthening |
| #759 F – Breathing exercises in sitting | #924 Seated spinal flexion with leg lift – breathing / mobilization | #924 Seated spinal flexion with leg lift – breathing / mobilization | #759 F – Breathing exercises in sitting | #759 F – Breathing exercises in sitting | #759 F – Breathing exercises in sitting | #296 Standing on tiptoes with eyes closed |
| #368 Hip opening in side-lying – gluteal/hip stretch | #759 F – Breathing exercises in sitting | #759 F – Breathing exercises in sitting | #368 Hip opening in side-lying – gluteal/hip stretch | #368 Hip opening in side-lying – gluteal/hip stretch | #368 Hip opening in side-lying – gluteal/hip stretch | #924 Seated spinal flexion with leg lift – breathing / mobilization |
| #146 Supine spinal rotation | #368 Hip opening in side-lying – gluteal/hip stretch | #368 Hip opening in side-lying – gluteal/hip stretch | #146 Supine spinal rotation | #146 Supine spinal rotation | #146 Supine spinal rotation | #759 F – Breathing exercises in sitting |
|  | #146 Supine spinal rotation | #146 Supine spinal rotation |  |  |  | #368 Hip opening in side-lying – gluteal/hip stretch |
|  |  |  |  |  |  | #146 Supine spinal rotation |

# Supplementary Information 3: Detailed description of secondary outcome measures

- Health-related quality of life (HRQoL) was assessed with the EuroQol-5 dimensions-5 level (EQ-5D-5L).[5] The EQ-5D-5L index value for the German population ranges from -0.205 (where 0 represents a health state equivalent to death and negative values indicate a health state worse than death) to 1 (indicating the best possible health state).[14]
- The Fatigue Severity Scale-7 (FSS-7) is used to evaluate fatigue. The seven-item version has better psychometric properties than the nine–item version.[7] Score: 1-7. The cut-off ≥ 4 was interpreted as indicative of fatigue.[13]
- The Hospital Anxiety and Depression Scale (HADS) is a valid and reliable tool to measure anxiety and depression and was also used in COVID-19 patients.[3] Score: 0-21 each for anxiety and depression. A score of >7 in each category was interpreted as clinically significant.[16]
- The generic World Health Organization Disability Assessment Schedule 2.0 (WHODAS-12) measures health and disability and comprises the categories cognition, mobility, self-care, getting along, life activities, and participation. It is reliable, widely used and has good internal consistency.[10] The total score was converted into a percentage and subsequently categorized: no (0-4%); mild (5-24%); moderate (25-49%); severe (50-95%); and complete (96-100%) disability.
- The Mini-BESTest with 14 items was used to measure dynamic balance. This test has good psychometric properties and is widely used in research and practice. The maximum score of 28 indicates the best balance. [2, 4]
- The grip strength was measured with a digital dynamometer (Kern MAP 801KS)at both hands in kilogram. Each hand was measured twice and the maximum value was used for further analyses.
- To evaluate cognitive function, the German version of the Montreal Cognitive Assessment (MoCA) was used. Its good reliability and validity was shown in different languages and in different patient groups. The maximum score is 30, whereby less than 26 points are indicative for mild cognitive impairment. [8] As the MoCA was repeated in every study visit, we used three different official versions, to avoid learning effects. For the telephone interviews (V5+V6) the telephone version of the MoCA was used, which also has good psychometric properties. For this version, the maximum score is 22 and the cut-off value of <18 was used to detect mild cognitive impairment. [15]
- To assess dyspnea, the modified medical research council dyspnea scale was used (score 0-4; 4=severest dyspnea).[1]
- The two-minute-walk-test was used to measure exercise capacity. Thereby, the distance is measured that can be walked on an even surface within two minutes. [9] This test was chosen instead of the six-minute-walk-test as it was too exhausting for many individuals with post COVID-19 symptoms to walk for six minutes. For the evaluation, we used portable mobility lab sensors (APDM Wearable Technologies Inc., Portland, US).
- The short form of DePaul Symptom Questionnaire was used to assess symptoms of myalgic encephalomyelitis and chronic fatigue syndrome.[12] This questionnaire was also used in a population with long COVID.[6] We used an own, not validated version, which we translated from English into German. For each of the 14 symptoms, the frequency and the symptom severity is rated on a five-point Likert scale (0-4), whereby higher values indicate higher frequency and severity of symptoms. To standardize the values to a 100-point scale, the frequency and severity scores of each symptom were averaged and multiplied with 25.[6]
- The modified COVID-19 Yorkshire Rehabilitation Screening (C19-YRS) was used in the self-report version.[11] It consists of the subscales symptom severity (score 0-30), functional disability (score 0-15), other symptoms (0-25) and the overall self-rated health score (0-10). The higher the score, the more severe the symptoms.
- Participants were provided with the smartwatches Fitbit Versa 2 / 3, unless they had their own high-quality smartwatch. These smartwatches were used to track resting heart rate and steps per day during four periods of 14 days each: the two weeks before the on-site therapy (V1-V2), during the two weeks of the therapy (V2-V3), the two weeks after the therapy (V3-V4) and two weeks around the first follow-up measurement V5.

| Supplementary Table 2 Number of available outcome data per study visit | | | | | | |
| --- | --- | --- | --- | --- | --- | --- |
|  | **Visit 1** | **Visit 2** | **Visit 3** | **Visit 4** | **Visit 5** | **Visit 6** |
| Fatigue-Severity-Scale 7 | 47 | 47 | 47 | 45 | 44 | 38 |
| Hospital and Anxiety Scale | 47 | 47 | 46 | 46 | 44 | 40 |
| Modified MRC Dyspnea Scale | 47 | 47 | 47 | 45 | 43 | 35 |
| Handgrip strength | 47 | 46 | 46 | 40 | N/A | N/A |
| EQ-5D-5L | 47 | 47 | 47 | 44 | 43 | 40 |
| WHODAS-12 | 47 | 47 | 47 | 46 | 44 | 39 |
| 2-minute-walking-test | 45 | 47 | 46 | 40 | N/A | N/A |
| MoCA | 47 | 46 | 46 | 40 | 43 | 40 |
| C19-YRS | 47 | 47 | 47 | 46 | 44 | 40 |
| Mini-BESTest | 47 | 47 | 46 | 41 | N/A | N/A |
| DePaul Questionnaire | 47 | 47 | 47 | 46 | 44 | 40 |
| C19-YRS = modified COVID-19 Yorkshire Rehabilitation Screening; EQ-5D-5l = EuroQol – 5 dimensions – 5 level; MoCA: Montreal Cognitive Assessment; Mini-BESTest: Mini Balance Evaluation Systems Test; WHODAS-12 = World Health Organization Disability Assessment Schedule 2.0 – 12 items | | | | | | |


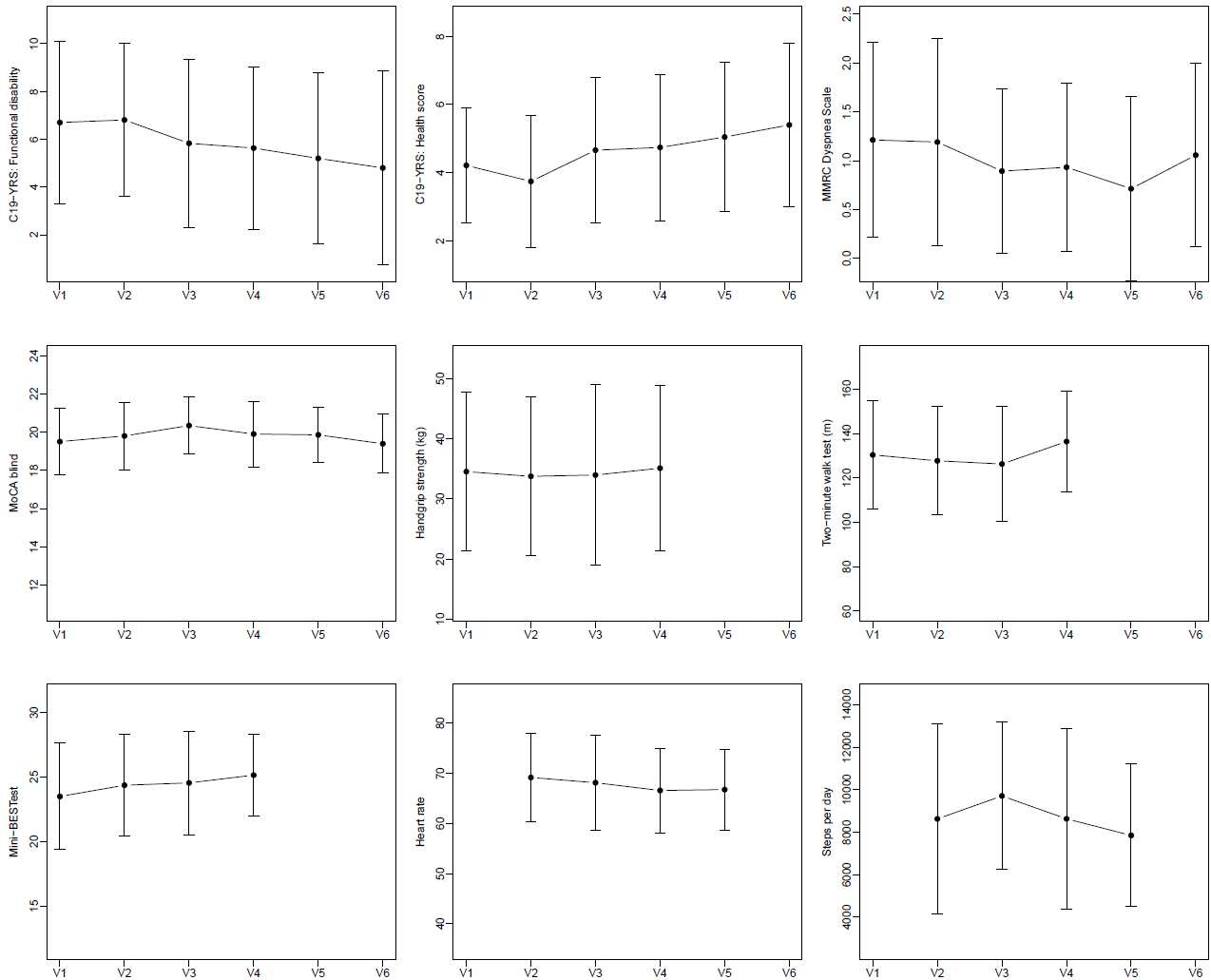


Supplementary Figure 1 Mean values of the assessments C19-YRS (functional disability and health score), MMRC dyspnea Scale , MoCA blind, handgrip strength, two-minute walk test, Mini-BESTest, and the resting heart rate and steps per day measured via smartwatch-based monitoring.


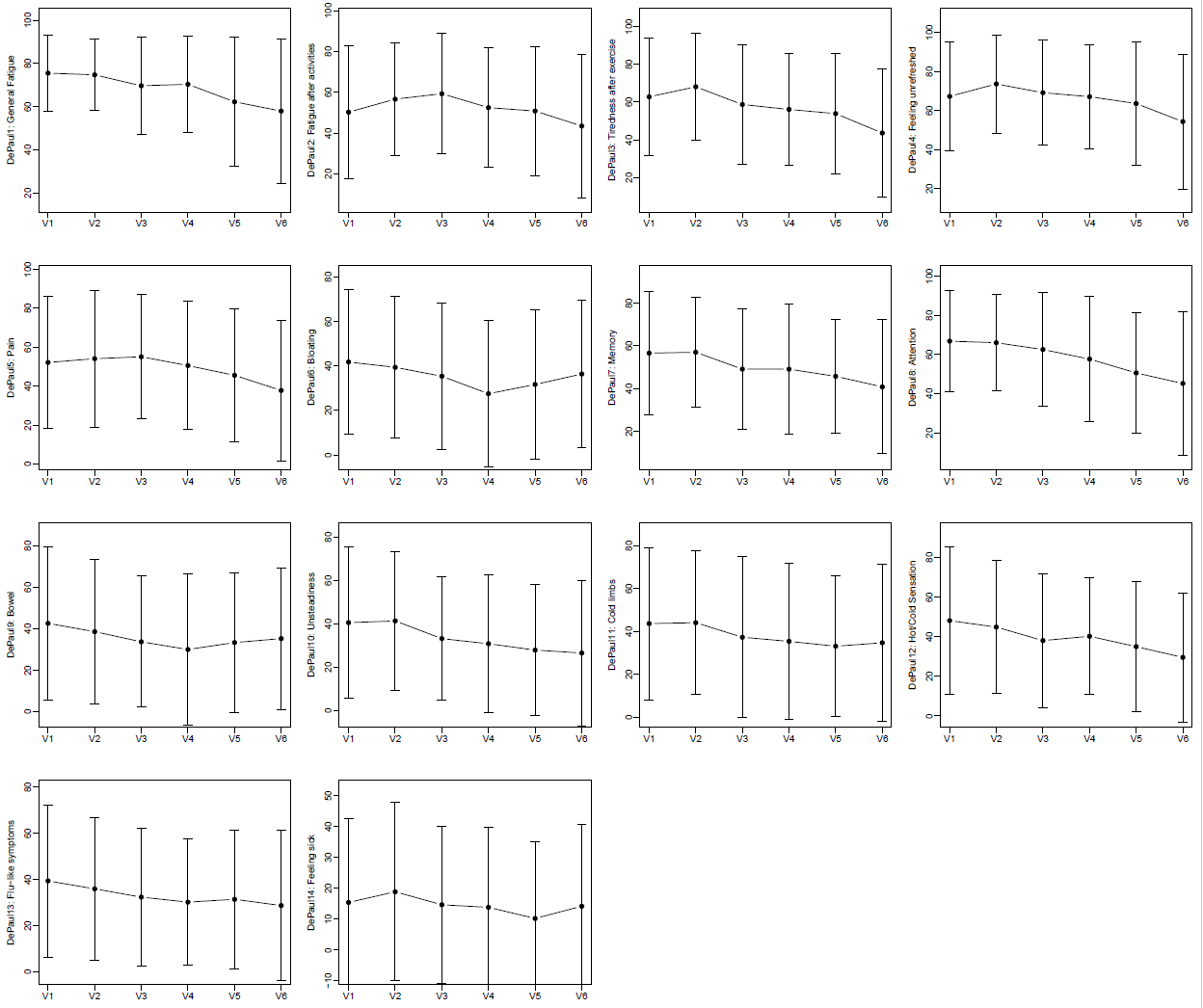


Supplementary Figure 2 Mean values of the 14 items of the short form of the DePaul Symptom Questionnaire

| Supplementary Table 3 Descriptive summary of the DePaul Symptom Questionnaire (short form) and results of the longitudinal mixed models. | | | | | | | | | | |  |
| --- | --- | --- | --- | --- | --- | --- | --- | --- | --- | --- | --- |
|  |  |  |  |  |  |  | **Change within…** | | | | |
|  | **Visit 1** | **Visit 2** | **Visit 3** | **Visit 4** | **Visit 5** | **Visit 6** | **Intervention period (V2-V3)** | **Two weeks  post-intervention (V3-V4)** | **Two months  post-intervention (V4-V5)** | **Six months post-intervention (V5-V6)** | |
| **1. Fatigue/extreme tiredness** | 75.5±17.5 | 74.7±16.6 | 69.7±22.7 | 70.4±22.4 | 63.6±28.6 | 63.8±29.4 | -5.05 (0.176) | 0.85 (0.822) | **-7.82 (0.004)** | -5.37 (0.164) | |
| **2. Next day soreness or fatigue after non-strenuous, everyday activities** | 50.3±32.5 | 56.7±27.6 | 59.3±29.5 | 52.5±29.2 | 52.0±31.0 | 47.8±34.0 | 2.66 (0.595) | -6.94 (0.171) | -1.28 (0.802) | -8.45 (0.102) | |
| **3. Minimum exercise makes you physically tired** | 62.8±30.9 | 68.1±28.2 | 58.7±31.5 | 56.1±29.8 | 55.1±31.2 | 48.1±32.5 | **-9.21 (0.021)** | -3.05 (0.451) | -1.92 (0.638) | **-10.98 (0.008)** | |
| **4. Feeling unrefreshed after you wake up in the morning** | 67.3±27.7 | 73.6±25.2 | 69.2±26.9 | 67.1±26.5 | 65.1±30.6 | 59.9±31.4 | -4.58 (0.282) | -0.72 (0.686) | -3.69 (0.391) | **-9.87 (0.025)** | |
| **5. Pain or aching in your muscles** | 52.1±33.8 | 54.1±35.1 | 55.1±32.0 | 50.5±32.9 | 46.6±33.9 | 41.6±35.6 | 1.44 (0.717) | -3.63 (0.360) | -5.44 (0.175) | **-8.35 (0.041)** | |
| **6. Bloating** | 41.8±32.6 | 39.4±31.7 | 35.4±32.8 | 27.5±32.8 | 32.4±33.5 | 40.0±32.4 | -3.99 (0.263) | -6.32 (0.081) | 3.49 (0.340) | 3.70 (0.316) | |
| **7. Problems remembering things** | 56.7±28.9 | 57.1±25.6 | 49.2±28.2 | 49.2±30.6 | 46.9±26.0 | 45.0±29.8 | **-7.00 (0.049)** | -0.53 (0.883) | -1.79 (0.619) | **-7.23 (0.047)** | |
| **8. Difficulty paying attention for a long period of time** | 66.8±25.6 | 66.0±24.7 | 62.5±29.0 | 57.6±32.0 | 51.7±30.1 | 49.7±35.4 | -3.71 (0.301) | -4.14 (0.253) | -5.70 (0.119) | **-8.16 (0.028)** | |
| **9. Irritable bowel problems** | 42.6±36.7 | 38.6±35.0 | 33.7±31.5 | 30.0±36.2 | 34.1±33.6 | 38.8±33.7 | -4.55 (0.286) | -2.43 (0.573) | 3.25 (0.453) | -0.11 (0.980) | |
| **10. Feeling unsteady on your feet, like you might fall** | 40.4±35.0 | 41.2±32.0 | 33.1±28.5 | 30.7±31.7 | 28.4±30.2 | 29.1±34.1 | **-7.69 (0.039)** | -1.92 (0.608) | -3.11 (0.406) | -2.45 (0.518) | |
| **11. Cold limbs (e.g. arms, legs, hands)** | 43.6±35.5 | 44.0±33.4 | 37.2±37.6 | 35.3±36.3 | 33.8±32.9 | 38.1±36.7 | **-7.08 (0.08)** | -2.09 (0.605) | -2.18 (0.591) | 0.83 (0.841) | |
| **12. Feeling hot or cold for no reason** | 48.1±37.0 | 44.8±33.6 | 38.0±33.9 | 40.2±29.5 | 35.8±32.6 | 32.5±32.7 | -6.08 (0.140) | 2.32 (0.573) | -5.18 (0.211) | -7.25 (0.084) | |
| **13. Flu-like symptoms** | 39.4±33.0 | 35.9±30.9 | 32.3±30.0 | 30.2±27.2 | 32.1±30.0 | 31.6±32.8 | -3.38 (0.352) | -1.56 (0.670) | 1.32 (0.719) | -4.82 (0.196) | |
| **14. Some smells, foods, medications, or chemicals make you feel sick** | 15.4±27.2 | 18.9±28.9 | 14.7±25.6 | 13.9±25.9 | 10.7±25.4 | 15.6±27.4 | -4.11 (0.224) | 0.45 (0.895) | -3.77 (0.272) | 1.83 (0.601) | |
| Data is presented as mean ± SD for descriptive statistics and as coefficients with p-values in brackets for the results of the longitudinal mixed model; Significant values are in bold. | | | | | | | | | | | |

| Supplementary Table 4 Effect of the risk factors age, sex, post-exertional malaise (PEM), cognition, subjective health score and interaction terms of time with PEM and cognition, respectively. Results of the risk factors are given as coefficient and 95% confidence intervals and the interactions term are given as the coefficients for each time point within the two vertical bars and the respective global p-value. | | | | | | | | |
| --- | --- | --- | --- | --- | --- | --- | --- | --- |
|  | **Age** | **Sex** | **PEM** | **Cognitive impairment** | **Subjective health score pre COVID-19** | **Interaction PEM** | **Interaction cognition** |  |
| EQ-5D-5L - Index value | 0.00 [0.00; 0.01] (pval=0.084) | **-0.11 [-0.2; -0.01] (pval=0.033)** | -0.07 [-0.21; 0.06] (pval=0.280) | -0.08 [-0.19; 0.02] (pval=0.119) | 0.04 [0; 0.08] (pval=0.057) | \|0.02; -0.03; -0.05; 0.02; 0.06\| (pval=0.800) | **\|-0.04; -0.11; -0.09; -0.07; 0.07\| (pval=0.038)** |  |
| Fatigue-Severity-Scale-7 | **-0.03 [-0.05; 0] (pval=0.024)** | 0.26 [-0.48; 0.99] (pval=0.498) | 0.85 [-0.18; 1.89] (pval=0.111) | 0.38 [-0.43; 1.19] (pval=0.354) | **-0.43 [-0.76; -0.09] (pval=0.015)** | \|-0.07; -0.4; -0.36; -0.19; -0.38\| (pval=0.958) | \|0.27; 0.38; 0.49; -0.10; 0.77\| (pval=0.253) |  |
| HADS |  |  |  |  |  |  |  |  |
| Anxiety | -0.07 [-0.14; 0] (pval=0.064) | 1.31 [-0.91; 3.53] (pval=0.249) | -0.47 [-3.57; 2.64] (pval=0.768) | -0.55 [-2.98; 1.88] (pval=0.654) | **-1.03 [-2.03; -0.04] (pval=0.047)** | \|-2.16; -1.22; -2.69; -2.6; -3\| (pval=0.161) | \|0.17; 1.39; 1.40; 1.25; 0.67\| (pval=0.455) |  |
| Depression | -0.04 [-0.11; 0.03] (pval=0.258) | 0.83 [-1.32; 2.98] (pval=0.448) | -0.91 [-3.92; 2.1] (pval=0.554) | 0.54 [-1.82; 2.89] (pval=0.654) | **-1.11 [-2.08; -0.15] (pval=0.028)** | \|-3.18; -1.41; -3.15; -1.88; -2.78\| (pval=0.056) | \|-0.87; 0.52; 0.23; -0.09; 0.17\| (pval=0.700) |  |
| WHODAS-12 Score, % | -0.13 [-0.43; 0.17] (pval=0.392) | **12.05 [2.80; 21.30] (pval=0.014)** | 9.23 [-3.74; 22.21] (pval=0.167) | 1.1 [-9.04; 11.24] (pval=0.830) | -4.14 [-8.3; 0.03] (pval=0.056) | \|-1.17; -1.96; -7.36; -11.97; -10.93\| (pval=0.076) | \|-4.58; 0.07; 3.13; 5.07; 0.17\| (pval=0.165) |  |
| C19-YRS |  |  |  |  |  |  |  |  |
| Symptom severity | -0.03 [-0.12; 0.06] (pval=0.523) | 2.56 [-0.21; 5.34] (pval=0.075) | 1.63 [-2.26; 5.52] (pval=0.412) | 2.79 [-0.26; 5.83] (pval=0.077) | **-1.40 [-2.65; -0.16] (pval=0.032)** | \|0.51; 0.05; -0.02; -3.08; -2\| (pval=0.404) | \|-0.52; 1.77; 1.43; -0.36; -0.25\| (pval=0.446) |  |
| Functional disability | -0.05 [-0.09; 0] (pval=0.072) | **2.28 [0.79; 3.78] (pval=0.004)** | **2.23 [0.14; 4.33] (pval=0.041)** | 0.21 [-1.43; 1.85] (pval=0.802) | **-0.78 [-1.45; -0.11] (pval=0.026)** | \|-0.38; -0.19; -1.74; -1.41; -1.03\| (pval=0.400) | \|-0.51; 0.31; 0.10; 0.24; -0.53\| (pval=0.771) |  |
| Health score | 0.01 [-0.02; 0.03] (pval=0.665) | **-1.04 [-1.83; -0.26] (pval=0.012)** | -0.7 [-1.8; 0.4] (pval=0.214) | -0.28 [-1.14; 0.58] (pval=0.530) | **0.38 [0.03; 0.73] (pval=0.040)** | \|0.12; 0.19; -0.57; 0.45; -0.76\| (pval=0.755) | \|0.06; -0.35; -0.15; -0.70; -0.93\| (pval=0.622) |  |
| Mod. Medical Research Council Dyspnea Scale | 0 [-0.01; 0.01] (pval=0.887) | 0.28 [-0.12; 0.69] (pval=0.171) | 0.35 [-0.21; 0.91] (pval=0.228) | 0.27 [-0.17; 0.71] (pval=0.238) | **-0.21 [-0.39; -0.03] (pval=0.028)** | \|0.31; 0.46; 0.52; 0.6; 0.55\| (pval=0.499) | \|0.22; -0.29; -0.31; 0.01; -0.15\| (pval=0.197) |  |
| MoCA blind | -0.01 [-0.03; 0.01] (pval=0.307) | 0.43 [-0.16; 1.01] (pval=0.156) | **0.99 [0.16; 1.81] (pval=0.022)** | -0.48 [-1.12; 0.17] (pval=0.150) | -0.11 [-0.37; 0.15] (pval=0.415) | **\|-0.47; -0.03; -0.71; -0.96; -2.67\| (pval=0.046)** | \|0.38; -0.39; -0.07; -0.8; -0.51\| (pval=0.371) |  |
| MoCA | -0.02 [-0.04; 0.01] (pval=0.192) | 0.33 [-0.43; 1.09] (pval=0.398) | **1.76 [0.71; 2.81] (pval=0.002)** | -0.55 [-1.38; 0.28] (pval=0.196) | -0.02 [-0.36; 0.32] (pval=0.925) | \|-0.4; -0.37; -1.07\| (pval=0.696) | \|0.62; -0.26; 0.17\| (pval=0.593) |  |
| Handgrip strength max., kg | **-0.30 [-0.42; -0.17] (pval<.001)** | **-21.87 [-25.73; -18.02] (pval<.001)** | -2.62 [-8.01; 2.77] (pval=0.345) | -3.3 [-7.53; 0.93] (pval=0.133) | -0.39 [-2.12; 1.34] (pval=0.659) | \|-2.17; -1.15; -2.32\| (pval=0.746) | \|1.26; 1.56; -1.07\| (pval=0.433) |  |
| 2-minute-walking-test - Distance | 0.28 [-0.15; 0.71] (pval=0.202) | -11.61 [-24.64; 1.42] (pval=0.087) | -5.47 [-23.7; 12.76] (pval=0.558) | 4.98 [-9.3; 19.27] (pval=0.496) | 4.12 [-1.74; 9.98] (pval=0.174) | \|9.93; 0.39; 6.49\| (pval=0.415) | \|1.32; 7.44; 2.01\| (pval=0.486) |  |
| Mini-BESTest | -0.04 [-0.1; 0.03] (pval=0.310) | -1.59 [-3.68; 0.51] (pval=0.143) | -0.92 [-3.85; 2.02] (pval=0.542) | -0.24 [-2.54; 2.06] (pval=0.837) | 0.84 [-0.11; 1.78] (pval=0.088) | \|-0.49; -0.55; -0.36\| (pval=0.885) | \|-0.14; 0.53; -0.17\| (pval=0.552) |  |
| C19-YRS = modified COVID-19 Yorkshire Rehabilitation Screening; EQ-5D-5l = EuroQol – 5 dimensions – 5 level; HADS = Hospital Anxiety and Depression Scale; MoCA= Montreal Cognitive Assessment; Mini-BESTest: Mini Balance Evaluation Systems Test; WHODAS-12 = World Health Organization Disability Assessment Schedule 2.0 – 12 items; Significant coefficients are in bold. | | | | | | | | |

# References

1. Bestall JC, Paul EA, Garrod R, Garnham R, Jones PW, Wedzicha JA (1999) Usefulness of the Medical Research Council (MRC) dyspnoea scale as a measure of disability in patients with chronic obstructive pulmonary disease. Thorax 54:581-586

2. Di Carlo S, Bravini E, Vercelli S, Massazza G, Ferriero G (2016) The Mini-BESTest: a review of psychometric properties. International journal of rehabilitation research Internationale Zeitschrift fur Rehabilitationsforschung Revue internationale de recherches de readaptation 39:97-105

3. Fernández-de-las-Peñas C, Rodríguez-Jiménez J, Palacios-Ceña M, de-la-Llave-Rincón AI, Fuensalida-Novo S, Florencio LL, Ambite-Quesada S, Ortega-Santiago R, Arias-Buría JL, Liew BXW, Hernández-Barrera V, Cigarán-Méndez M (2022) Psychometric Properties of the Hospital Anxiety and Depression Scale (HADS) in Previously Hospitalized COVID-19 Patients. International Journal of Environmental Research and Public Health 19:9273

4. Franchignoni F, Horak F, Godi M, Nardone A, Giordano A (2010) Using psychometric techniques to improve the Balance Evaluation Systems Test: the mini-BESTest. J Rehabil Med 42:323-331

5. Janssen MF, Bonsel GJ, Luo N (2018) Is EQ-5D-5L Better Than EQ-5D-3L? A Head-to-Head Comparison of Descriptive Systems and Value Sets from Seven Countries. PharmacoEconomics 36:675-697

6. Jason LA, Dorri JA (2022) ME/CFS and Post-Exertional Malaise among Patients with Long COVID. Neurology international 15:1-11

7. Johansson S, Kottorp A, Lee KA, Gay CL, Lerdal A (2014) Can the Fatigue Severity Scale 7-item version be used across different patient populations as a generic fatigue measure--a comparative study using a Rasch model approach. Health and quality of life outcomes 12:24

8. Nasreddine ZS, Phillips NA, Bedirian V, Charbonneau S, Whitehead V, Collin I, Cummings JL, Chertkow H (2005) The Montreal Cognitive Assessment, MoCA: a brief screening tool for mild cognitive impairment. J Am Geriatr Soc 53:695-699

9. Pin TW (2014) Psychometric properties of 2-minute walk test: a systematic review. Archives of physical medicine and rehabilitation 95:1759-1775

10. Saltychev M, Katajapuu N, Bärlund E, Laimi K (2021) Psychometric properties of 12-item self-administered World Health Organization disability assessment schedule 2.0 (WHODAS 2.0) among general population and people with non-acute physical causes of disability - systematic review. Disability and rehabilitation 43:789-794

11. Sivan M, Preston N, Parkin A, Makower S, Gee J, Ross D, Tarrant R, Davison J, Halpin S, O'Connor RJ, Horton M (2022) The modified COVID-19 Yorkshire Rehabilitation Scale (C19-YRSm) patient-reported outcome measure for Long Covid or Post-COVID-19 syndrome. Journal of medical virology 94:4253-4264

12. Sunnquist M, Lazarus S, Jason LA (2019) The development of a short form of the DePaul Symptom Questionnaire. Rehabilitation psychology 64:453-462

13. Valko PO, Bassetti CL, Bloch KE, Held U, Baumann CR (2008) Validation of the fatigue severity scale in a Swiss cohort. Sleep 31:1601-1607

14. van Hout B, Janssen MF, Feng YS, Kohlmann T, Busschbach J, Golicki D, Lloyd A, Scalone L, Kind P, Pickard AS (2012) Interim scoring for the EQ-5D-5L: mapping the EQ-5D-5L to EQ-5D-3L value sets. Value in health : the journal of the International Society for Pharmacoeconomics and Outcomes Research 15:708-715

15. Zietemann V, Kopczak A, Muller C, Wollenweber FA, Dichgans M (2017) Validation of the Telephone Interview of Cognitive Status and Telephone Montreal Cognitive Assessment Against Detailed Cognitive Testing and Clinical Diagnosis of Mild Cognitive Impairment After Stroke. Stroke 48:2952-2957

16. Zigmond AS, Snaith RP (1983) The Hospital Anxiety and Depression Scale. Acta Psychiatrica Scandinavica 67:361-370
